# The stability and modulation of if-then rules versus prospective planning in movement selection under dual-tasking conditions

**DOI:** 10.1038/s41598-024-81630-5

**Published:** 2025-01-08

**Authors:** S. E. M. Stoll, A. Wenzel, B. Hitzler, J. Randerath

**Affiliations:** 1 Department of Psychology, University of Konstanz, Konstanz, Germany; 2 Lurija Institute for Rehabilitation Science and Health Research, Kliniken Schmieder, Allensbach, Germany; 3 Department of Developmental and Educational Psychology, Faculty of Psychology, University of Vienna, Vienna, Austria; 4 Clinical Neuropsychology and Neuropsychological Psychotherapy, Institute of Psychology, University of Regensburg, Regensburg, Germany

**Keywords:** Prospective planning, Implementation intentions, Dual-tasking, Movement selection, Psychology, Human behaviour

## Abstract

Two approaches to movement selection, if-then rules versus prospective planning, were investigated. Studies have shown that the rule-based approach leads to more efficient movement selection than the plan-based approach, though the resulting movements are the same. This dual-tasking study investigates two hypotheses explaining this discrepancy: The efficiency hypothesis states that the rule-based approach to movement selection is more efficient, and its advantage over the plan-based approach increases under any kind of enhanced task demands. The dual-mechanisms hypothesis states that the two approaches to movement selection are based on distinct mechanisms and that they can be differentially affected by secondary tasks. Forty-eight participants selected a grip in order to comfortably rotate a dowel to a target position using the rule- or the plan-based approach. Four dual-tasking conditions were added: Identifying the activity underlying an object-interaction sound, identifying and categorizing the object underlying an object-interaction sound, identifying one of four words that does not match the others semantically, and solving calculations. The rule-based approach led to more efficient movement selection in most dual-tasking conditions. However, its advantage disappeared when the secondary task was to identify and categorize objects. These results strengthen the dual-mechanisms hypothesis, and possible factors distinguishing the approaches are discussed.

**Supplementary Information:**

The online version contains supplementary material available at 10.1038/s41598-024-81630-5.

## Introduction

When people navigate and interact with objects, they perform complex, demanding tasks. A key step in acting effectively is selecting basic movements suited to action goals. For example, when opening an oven door, one must choose a grip that allows opening the door without getting burned.

Different approaches to movement selection may apply depending on the context or action goal. A series of motor-cognitive studies^1–6^ investigated two approaches to movement selection. The so-called rule-based approach presents a prespecified rule with an if-then sentence in which grip choice is based on fixed stimulus-response mappings. The “if” part of the sentence defines a certain stimulus configuration, and the “then” part of the sentence specifies the associated action (e.g., the adequate grip). In the other approach, the so-called plan-based approach, grip selection is based on flexible stimulus-response mappings, using prospective motor planning, i.e., anticipating the entire movement, including its end position, to select the initial grip.

If-then rules have been discussed for several years as “implementation intentions”, a term introduced by Gollwitzer^7^. Studies have shown that these if-then rules facilitate the initiation of desired behaviors across different conditions and populations^8^. In contrast, movements based on flexible stimulus-response mappings require prospective planning, considering both the action goal and the changing positions of the body when selecting the initial movement. For instance, one would choose a different grip on a cabinet door, depending on its opening direction (e.g., side versus downwards) and position (e.g., below versus above). Most people select an initial position that leaves them in a comfortable position when the demands for aiming are highest^9^. Since this was first observed at the end of the movement, this effect is termed the end-state comfort effect^10,11^.

The rule/plan motor cognition (RPMC) paradigm investigates rule- and plan-based approaches to movement selection in a single controlled setting. Participants select a grip that enables a comfortable rotation of a dowel to a target. While stimulus material, manipulation of the environment, and visually observable movements are similar throughout the experiment, only the instructions for selecting the grip differ between the plan- (“Perform the movement as comfortably as possible”) versus rule-based approaches (e.g. “If the target is green, then place your thumb towards the green cue light on the dowel”). Participants repeatedly showed shorter reaction times and fewer errors when selecting movements with the rule-based approach compared to the plan-based approach, indicating higher efficiency of the rule-based approach. This “rule-based efficiency advantage” was replicated in the RPMC paradigm across multiple studies^2–4,6^. However, the underlying mechanisms that serve this rule-based efficiency advantage remain unclear, with two competing hypotheses under consideration. The first hypothesis states that both approaches share a single mechanism and that the rule-based approach leads to more efficient use of this mechanism (efficiency hypothesis). If the rule-based efficiency advantage increases under any kind of enhanced task demands (e.g., pantomiming, interleaved experimental design, secondary tasks), the efficiency hypothesis is supported. The second hypothesis states two distinct mechanisms for the two approaches, one supporting rule-based movement selection and the other supporting plan-based movement selection (dual-mechanisms hypothesis). Conversely, if external factors (e.g., preceding tasks, psychosocial stress, aging, secondary tasks, etc.) affect the rule- versus the plan-based approach to movement selection differently, the dual-mechanisms hypothesis is supported.

### The efficiency hypothesis

Scheib et al.^4^ investigated the rule- and plan-based approaches in settings of varying difficulty and found that the rule-based efficiency advantage (defined as the performance difference between the rule- and the plan-based approach to movement selection) increased when enhanced task demands are imposed (e.g., pantomiming object interactions versus real object interactions). This suggests that when task demands may be elevated, there seems to be a relatively stronger negative impact on the potentially less efficient plan-based approach. Drift diffusion analyses of reaction times and error rates further supported increased efficiency in the rule-based approach: The information accumulation was more efficient in trials solved with the rule-based approach, while other drift diffusion parameters, such as non-decision time and boundary separation, did not differ between the two approaches, supporting the idea of a shared mechanism that is used at different levels of efficiency. The efficiency hypothesis is also supported by Randerath et al.^3^ who used fMRI to study the neural correlates of rule- and plan-based movement selection. The authors did not find clearly distinct activation patterns for the plan- versus the rule-based approach. Left parietal activation was prominent during the plan-based approach, but for the rule-based approach, there was no similarly prominent region. The results can be explained by shared neural structures underlying both approaches, with the rule-based approach to movement selection being perhaps more efficient. However, it remains unclear whether the rule-based approach simply requires less activation while using the same network or whether the two approaches use a similar network but in different ways (e.g., regarding timing).

### The dual-mechanisms hypothesis

The dual-mechanisms hypothesis states that the mechanisms subserving the rule- versus plan-based approach are at least partly distinct. When Randerath, et al.^2^ initially introduced the RPMC paradigm, they interpreted their findings as two distinct routes that lead to the same action. In a repetition-priming study, they observed the general rule-based efficiency advantage and a facilitation of action initiation in repeated trials, both in terms of shorter reaction times. Interestingly, the authors reported a significantly larger repetition effect for the plan-based than for the rule-based approach. They concluded that the two approaches might use different routes with the plan-based approach being more susceptible to external influences (e.g., repetition of stimuli) than the rule-based approach. Stoll, et al.^6^ further supported this interpretation by examining the RPMC paradigm under psychosocial stress. Regression analyses revealed that the rule-based approach remained robust against the stress effects. However, performance fluctuations in the plan-based approach could best be explained by stress-induced changes in heart rate variability, i.e., in the activity of the autonomic nervous system. Again, the plan-based approach appeared to be susceptible to external influences, while the rule-based approach was not. This suggests that there might be at least partly separate mechanisms subserving the rule- and plan-based approaches. A recent study by Scheib, et al.^5^ further supports the dual-mechanisms hypothesis. They found that the reaction time differences between the plan- and rule-based approaches increased with the advancing age of participants, but the rule-based efficiency advantage vanished in the oldest group aged 71–90 years. Some older participants maintained the rule-based efficiency advantage, while others favored the plan-based approach. This led the authors to conclude that the two approaches likely rely on different mechanisms, which aging may affect differently, resulting in greater inter-individual differences in advantages of the rule- versus plan-based approach.

### The current study

**Fig. 1:**
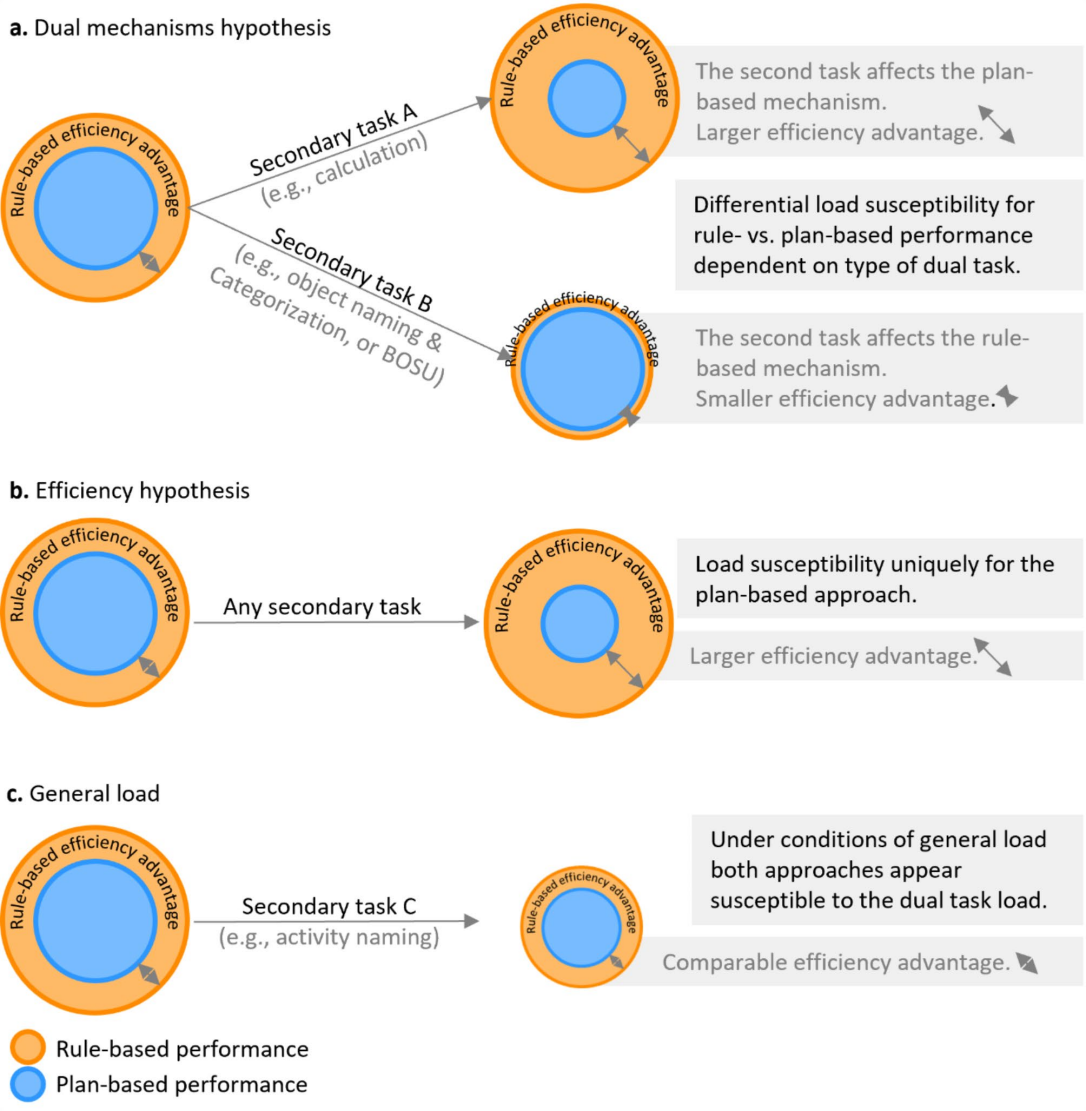
Possible interrelations between performances of rule- and plan-based approaches under dual-task load. Blue circles represent the performance in the plan-based approach, while orange circles represent the performance in the rule-based approach. Larger circles refer to better performance. The non-overlapping parts and arrows reflect the rule-based efficiency advantage at baseline (circles on the left) and under dual-task load (circles on the right), that is defined as the difference between the rule- and the plan-based approach.

The current study aimed to investigate further evidence for separate mechanisms in rule- versus plan-based movement selection approaches by combining the RPMC paradigm with different secondary tasks in a dual-task design. According to Wickens’^12^ multiple resource theory, conducting tasks simultaneously might lead to greater interference, when tasks draw upon overlapping resources. For example, listening to a podcast and reading might lead to interference since these are both verbal tasks, competing for common perceptual resources. In contrast, listening to a podcast and driving a car might lead to weaker interference because those activities (verbal versus spatial) draw from different perceptual resources. If the mechanisms in the RPMC paradigm are distinct, different secondary tasks should affect the rule- and plan-based approaches separately by selecting secondary tasks that interfere exclusively with the plan- or rule-based approach. Compared to a single-task rule-based over plan-based efficiency advantage, quantified as the performance difference in the rule- versus plan-based approach, a secondary task should then specifically reduce or enlarge this efficiency advantage (Fig. 1a). In contrast, if the efficiency hypothesis applies, i.e., both approaches are fed by similar resources but the rule-based approach uses them more efficiently, the following would be expected: Similar to the finding that increased task-difficulty affects the plan-based approach more strongly than the rule-based approach^4^, the rule-based efficiency advantage would increase under any secondary task (Fig. 1b). Finally, it might be possible that a secondary task equally loads on the rule- and plan-based approaches. This means that performance might be affected in both approaches, but the rule-based efficiency advantage remains unchanged (Fig. 1c).

#### Selection of the secondary tasks

Secondary tasks were selected based on their potential to interfere with either the rule- or plan-based approach to movement selection. To interfere with the plan- versus the rule-based approach, it is necessary to include secondary tasks that likely draw from similar mechanisms as the plan- or the rule-based approach, respectively. Such secondary tasks may involve factors important for object interactions, such as object knowledge or action knowledge, or they may more generally load on verbal or spatial working memory mechanisms. Auditive tasks, including the processing of sounds, can be easily added to action-related tasks without putting a direct load on the visual or motor system.

Identifying the activity underlying object-interaction sounds was selected as one of four secondary tasks. Previous studies showed that sounds associated with limb-related actions, such as clapping hands, seem to be processed in a left-lateralized network, including premotor, inferior parietal^13^, and fronto-parietal^14^regions. Also from a linguistic perspective, studies have emphasized the role of the left parietal cortex^15,16^, as well as frontal areas, like the prefrontal cortex^17^ and the pre-SMA^16^, in processing action knowledge. Priming participants to focus on the activity aspect of object-interaction sounds might interfere with the plan-based approach since Randerath et al.^3^ found a prominent role of the left parietal cortex in the plan-based approach, which might overlap with regions involved in identifying activities from object-interaction sounds.

Furthermore, it is hypothesized that the secondary task of identifying activities from object-interaction sounds could also interfere with the rule-based approach. On the one hand, activations in the prefrontal cortex have been associated with “if-then”-based instructions^18^, overlapping with regions associated with action knowledge^17^. On the other hand, there might be a semantic component in the rule-based approach that relies on the storage and repeated retrieval of the rule’s wording. Literature shows that fronto-temporal areas are relevant for processing rules^19^, and different areas of the temporal lobe have been discussed as neural substrates for various semantic tasks^20^, such as processing tool-related action sounds^21^ or identifying objects^22^. Therefore, identifying activities from object-interaction sounds might interfere with the rule-based approach due to processing overlaps in frontal and temporal regions.

In contrast, the mechanism underlying the rule-based approach might be selectively disturbed by the task of identifying and categorizing objects from object-interaction sounds. Processing object knowledge has been linked to left inferior temporal regions^17^, which could overlap with regions involved in the rule-based approach. Viewed from a multiple resource theory^12^ perspective, it was aimed to strain specifically the rule-based approach by loading the semantic resource with the secondary task of inferring and categorizing objects from object-interaction sounds.

Aside from the two secondary tasks involving object-interaction sounds, which were newly developed for the present study, the RPMC paradigm was combined with a working memory task that involved rather spatial (calculation of two-digit numbers) or rather verbal (BOSU categorical semantic test) material. Regarding calculation, the bilateral parietal lobes that serve the internal representation of numbers have been discussed^23,24^. This suggests that the secondary task calculation might interfere with the plan-based approach, which is primarily supported by the left, but also by the right parietal lobe^3^. Weaker performance in the semantic test BOSU is associated with temporally located lesions^25^. Therefore, solving semantic test trials from BOSU might lead to interference effects with the rule-based approach, which may rely on a semantic resource.

#### Physiological effects of dual-task load

Furthermore, heart rate variability (HRV) was assessed throughout the experiment. In a former study^6^, it was found that changes in the HRV variable RMSSD were predictive of performance changes in plan-based movement selection. This suggests that the processes involved in plan-based movement selection are sensitive to changes in the parasympathetic nervous system (PNS) due to stress. With respect to a meta-analysis by Becker, et al.^26^ stating that a decrease in PNS activity may also be observed in multi- and dual-tasking compared to single-tasking, the HRV variable RMSSD was included to evaluate the impact of dual-tasking on the participants’ stress system. Moreover, since RMSSD appears sensitive to dual-tasking load^26,27^ and the plan-based but not the rule-based approach was sensitive to changes in RMSSD in a former study^6^, the RMSSD may be a promising variable to investigate differences between the rule- and the plan-based approaches.

### Hypotheses

In the current experiment, the rule- versus plan-based approach to movement selection was investigated in a single-task condition and in combination with the dual-task conditions calculation, BOSU, listening to object-interactions, and identifying the activity or identifying and categorizing the object. The aim of the study was to shed light on the question of whether the rule-based efficiency advantage can be explained best by the efficiency hypothesis or the dual routes hypothesis.

1. If the efficiency hypothesis is correct, the rule-based efficiency advantage (defined as the performance difference between the rule- and the plan-based approach) is expected to be larger in the dual-task conditions compared to the single-task condition. According to the efficiency hypothesis, this should be due to a stronger impact of dual-task load on the plan-based approach than on the rule-based approach.

In case the dual routes hypothesis is correct, the following hypotheses are formulated:

(2) The rule-based efficiency advantage is expected to decrease in the object and category naming condition and in the semantic BOSU condition.
(3) The rule-based efficiency advantage is expected to increase in the calculation condition.

## Methods

### Participants

Forty-eight adults, free from any acute psychiatric or neurological disorder, participated in this study using a within-subject design. An analysis with G-Power^28^, based on data from a former study^6^, indicated that a difference between trials solved with the rule- versus plan-based approach could be reliably detected at a sample size of 14 participants (two-tailed, power = 0.95, α = 0.05, effect size dz = 1.06). However, for a counterbalanced design that considers the sequence of single- and dual-task blocks, the two color options for stimulus presentation, as well as the sequence of specific trials, it was necessary to include *N*= 48 participants. The participants were between 18 and 32 years old (M = 21.92 years, SD = 2.84 years) and 11 of them were male (22.92%). All of them were right-handed according to the method described by Salmaso and Longoni^29^ (lateralization quotient ≥ 66.67). The study was conducted in accordance with the Declaration of Helsinki, approved by the Ethics Committee of the University of Konstanz (#15/2020) and all individuals gave informed consent before participating. Data were collected from March until June 2022.

### Material

**Fig. 2:**
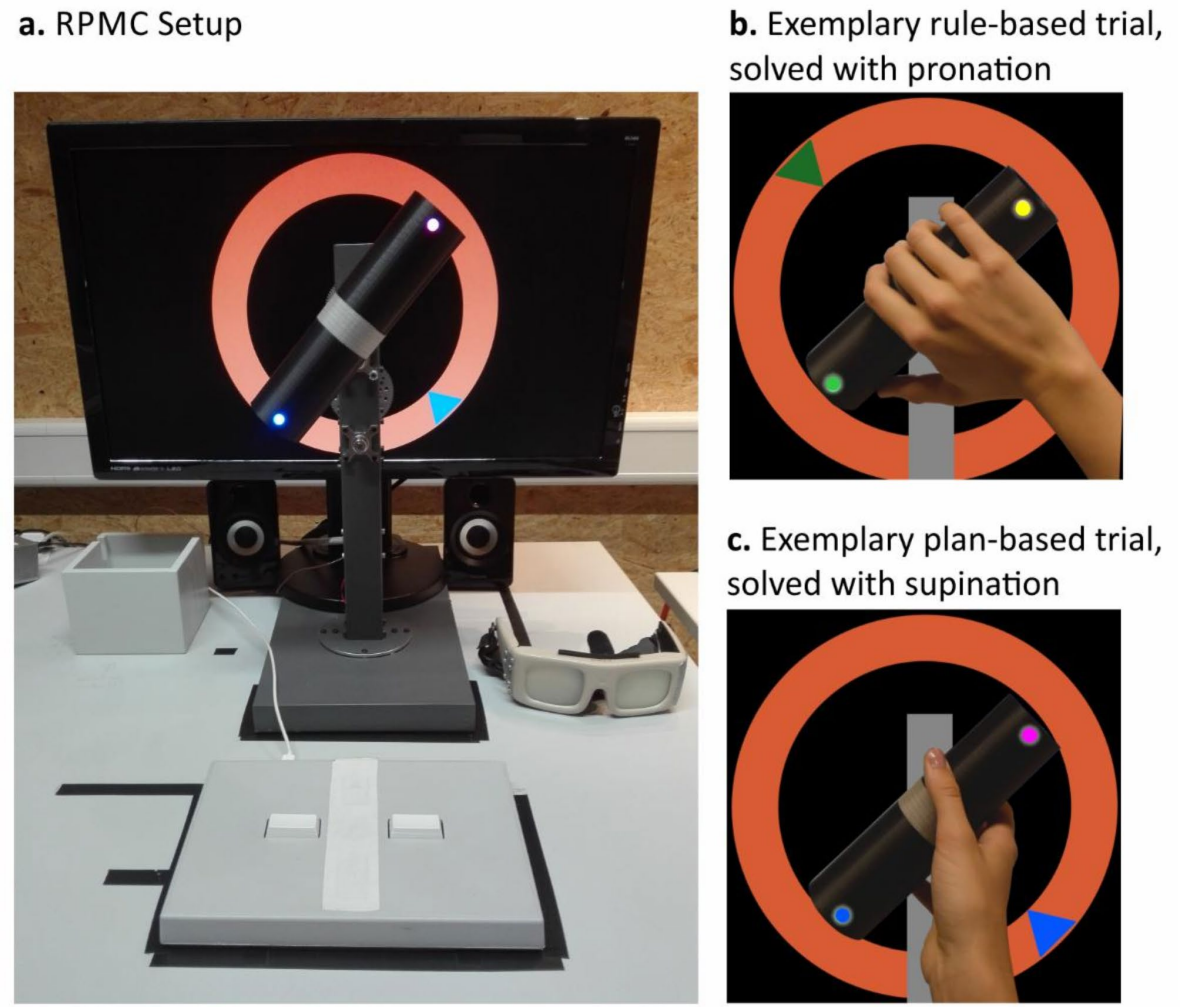
Display of the RPMC Setup and exemplary solutions to trials solved according to the rule- (exemplary instruction: “If the arrow is green, then I place my thumb on the side of the dowel with the green dot.”) (**b**) and the plan-based (**c**) approach (instruction: “I will carry out the movement as comfortably as possible”). (**a**) Picture of the RPMC setup, including the custom-built turning apparatus in front of the computer screen, response pad (front and center) and shutter goggles (right); adapted from Stoll, et al.^6^. (**b**) Picture of an RPMC trial which has been solved according to the rule-based approach with a pronated grasp: participants follow the if-then rule. (**c**) Picture of an RPMC trial which has been solved according to the plan-based approach with a supinated grasp: participants prospectively plan the movement and select a grip that allows them to perform the movement as comfortably as possible.

The experiment ran on a Windows 10 PC with the experimental software SuperLab 5 (Cedrus Corporation, San Pedro, CA, USA). Participants used a custom-built apparatus consisting of a rotating dowel on a pedestal to perform a rotation movement. LEDs were installed on the dowel’s right and left ends (Fig. 2a). Reaction and movement times were assessed with an RB-540 response pad (Cedrus Corporation, San Pedro, CA, USA) and shutter goggles that could switch between opaque (closed) and transparent (open) (PLATO Visual Occlusion Spectacles by Translucent Technology Incorporated, Canada).

A Polar H10 heart rate sensor (Polar Electro GmbH Deutschland, Germany), which was mounted around the participants’ chests, was used to measure the participants’ heart rate. Heart rate was tracked throughout the experiment, supported by the Heart Rate Variability Logger App^30^.

### Stimuli

#### RPMC experiment

Visual cues were presented on a computer screen. White arrows pointing to the right or to the left on a black background indicated which hand should be used to solve the following trial. Pink, blue, green, or yellow triangles on an orange ring on a black background served as stimuli for the target position of the dowel (Fig. 2b , c).

#### Secondary tasks

Four different secondary tasks were used: Identifying activities from object-interaction sounds, identifying and categorizing objects from object-interaction sounds, calculation, and BOSU.

The same 16 object-interaction sounds were used for the task of identifying the underlying action and to identify and categorize the underlying object. The sounds included interactions with everyday objects (e.g., writing with a pencil) and musical instruments (e.g., playing the recorder), lasting approximately 5000 ms each (for an overview of all sounds and their corresponding activities, objects, and categories, see Table 1).

**Table 1:** List of object-interaction sounds used in the experiment.

Activity	Object (category)
Hammering	Hammer (tool)
Sawing	Saw (tool)
Drilling	Drill (tool)
Chopping	Axe (tool)
Playing the xylophone	Xylophone (musical instrument)
Playing the recorder	Recorder (musical instrument)
Playing the guitar	Guitar (musical instrument)
Playing the accordion	Accordion (musical instrument)
Playing dice	Dice (household)
Teeth brushing	Toothbrush (household)
Pouring	Liquid (household)
Unzipping	Zipper (household)
Clicking	Computer Mouse (office)
Using the stapler	Stapler (office)
Writing	Pencil (office)
Typing	Keyboard (office)

For the calculation secondary task, 16 summations were selected from the stimulus pool of a study by Blini, et al.^31^. Calculations were voice-recorded and edited, with the final recordings lasting approximately 3300 ms.

Sixteen items from the BOSU^32^ were voice-recorded and served as secondary tasks. During the BOSU task, participants listened to four words and had to identify one of the four words that did not match the others semantically. Again, the recordings were edited to last approximately 3300 ms.

#### Generation of object-interaction sounds

All sounds used in the present study were recorded with a Zoom H4n recording device (Sound Service GmbH, Germany) and were edited with the sound processing program Audacity (iWeb Media Ltd., Malta). Initially, 42 sounds were recorded. The recognizability of the interaction sounds was evaluated in an online experiment using lab.js^33^ and Open Lab^34^. Forty subjects participated in the online study (20% male, mean age = 22.63 years). Only for the purpose of this pre-study, and in order to equally represent different kinds of object-interactions, sounds were categorized into sounds of musical instruments, tools, household or office items. After exclusion of sounds with ambiguous categorical affiliation (e.g., the sound of a bell: category household versus musical instrument), the four sounds that were recognized most often in each category were selected for the experiment (Table 1).

### Procedure

Upon arrival at the laboratory, participants were greeted by the experimenter and instructed to mount the heart rate sensor. This was followed by a 5-minute heart rate baseline assessment, where participants were instructed to sit comfortably in a chair and look at a white fixation cross on a black screen. Next, the movement selection experiment was conducted. In the experiment, participants performed 256 trials of the rule/plan motor cognition paradigm (RPMC). The trials were presented in 16 blocks of 16 trials each, either in a single-task condition or in combination with a secondary task (dual-task condition). The single- and dual-task blocks were presented alternately. A short break occurred after the first half of the experiment; in the second half, the exact same single- and dual-task trials were presented in reverse order. Participants were presented with trials that had to be solved using either the plan- or the rule-based approach in a randomized sequence. The sequence of the blocks was counterbalanced between subjects based on parameters such as trial order, beginning with dual- or single-task, whether the secondary task was intended to interfere with the rule- or plan-based approach, and whether the secondary task was based on object-interaction sounds or calculation and BOSU. This resulted in 48 different sequences, meaning that every subject completed the experiment, including single- and all kinds of dual-tasks, in their individual design. The duration of the session was about 2.5 h.

#### The RPMC in the single-task condition

**Fig. 3:**
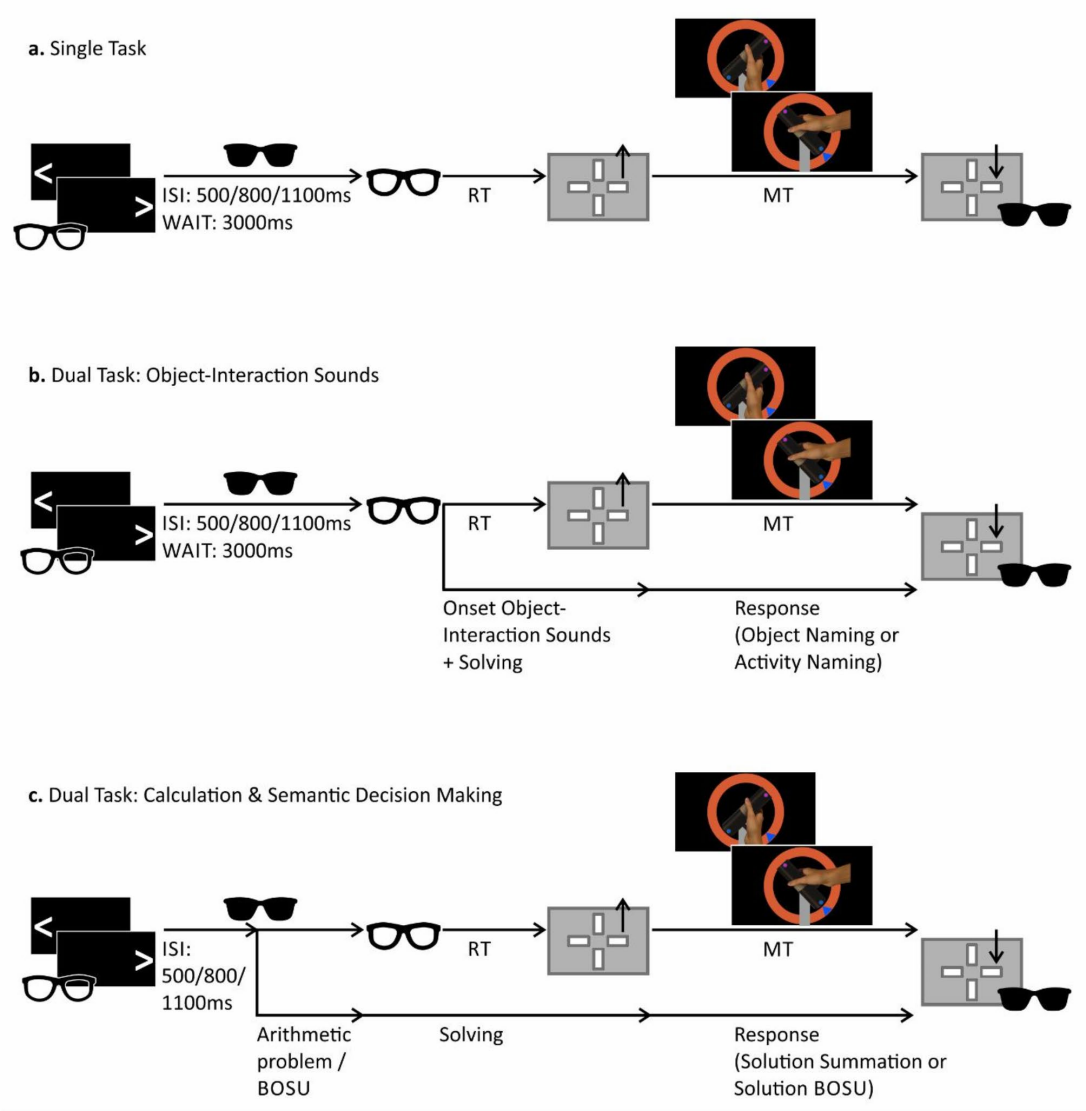
Schematic illustration of the RPMC protocol in the (**a**) single-task version, (**b**) dual-task version with object-interaction sounds, and (**c**) dual-task version with calculation and BOSU. Black rectangles with white arrows represent the visual cue indicating which hand to use in the upcoming trial; grey boxes with arrows pointing up and down represent releasing (up) and pressing (down) the response pad’s button; regular glasses represent opened shutter goggles, while sunglasses represent closed shutter goggles; RT = reaction time; MT = movement time; ISI = inter-stimulus interval; WAIT = waiting period; BOSU = Bogenhausener Semantik Untersuchung (semantic test).

In the single-task version (Fig. 3a), the RPMC trial began with the shutter goggles closed, and the participants pressed the left and right buttons of the response pad with their left and right hands. The goggles opened, and a visual cue indicated whether the subjects should solve the next trial with the right or left hand. When the goggles were closed again, the apparatus automatically turned the dowel to the starting position of the current trial.

The goggles were opened after a variable inter-stimulus interval and a fixed waiting period. Color cues indicated whether the trial needed to be addressed using the plan-based or rule-based approach. For the plan-based approach, participants were instructed to carry out the movement as comfortably as possible (“I will carry out the movement as comfortably as possible”). In contrast, for the rule-based approach, participants were instructed to place their thumb toward the LED that would show the same color as the target (e.g., “If the arrow is yellow, then I place my thumb on the side of the dowel with the yellow dot. If the arrow is green, then I place my thumb on the side of the dowel with the green dot.”). The color cues for the specific approaches (pink/blue or yellow/green) were counterbalanced between subjects.

Then, the participants selected the adequate movement by choosing one of two possible grips to hold the dowel, either a supination or a pronation (Fig. 2b + c). Next, the participants released the button of the response pad, grasped the dowel, and rotated it by 90° to a given target position, using the previously indicated hand. This means that the participants had to align the color-matching LED with the target arrow. Next, the participants pressed the button on the response pad again, triggering the closing of the goggles. The experimenter instantly assessed whether the participants produced an adequate grip, i.e., one resulting in a biomechanically comfortable end-position or one that was in accordance with the rule-based approach. Afterward, the experiment moved on to the following trial. Participants were not instructed to perform the task as fast as possible, but they were thoroughly instructed to accurately perform the movement.

Reaction time was assessed from the moment the participants saw the current trial (i.e., when the goggles opened) to the moment the participants began the movement (i.e., released the button). Movement time was measured from button release to button press. Participants were instructed to begin the movement after deciding how to grasp the dowel. This means that the reaction time, rather than the movement time, is used to reflect the cognitive movement selection period.

#### The RPMC in the dual-task condition

The protocol of the RPMC’s dual-task condition was similar to that of the single-task condition with the addition of the secondary tasks. In all dual-task conditions, participants were instructed to give their verbal responses in the later phase of the movement, i.e., while rotating the dowel. This ensured that the motor and verbal responses were separated as much as possible from the planning and solving phases, while the cognitive processes for movement planning and secondary task solving overlapped.

Dual-task trials including object-interaction sounds differed slightly from dual-task trials including calculations or BOSU. This was due to the consideration that the timing of mentally solving the secondary task is decisive in order to interfere with the movement selection process. Since the type of input was different between calculation/BOSU (discrete information: two summands, four words; before solving the task, participants must have heard the complete information) and object-interaction sounds (continuous information; task can be solved while listening to the information), the timing of the dual-task presentation varied between calculation/BOSU trials and object-interaction sound trials (see Fig. 3b , c).

Object-interaction sounds for both, identification of activities and identification and categorization of objects, were presented after a variable inter-stimulus interval and a fixed waiting period. The object-interaction sounds consisted of a continuous information unit, allowing the participants to start solving the secondary task as soon as the sound began. Therefore, the auditory input of the object-interaction sounds was presented concurrently with the RPMC trial, i.e., after both the inter-stimulus interval and the waiting period (Fig. 3b). This means that participants were confronted simultaneously with the current RPMC constellation and the object-interaction sound. The participants were instructed to select the adequate grip while mentally solving the secondary task, by imagining engaging in the activity reflected by the sound or by visualizing every detail of the object involved in the object-interaction sound. In the object-naming task, participants also had to come up with a category into which the object would fit (Table 1; examples of categories are given in parentheses).

Calculation and BOSU tasks were presented auditorily and directly after the inter-stimulus interval, i.e., during the waiting period, just before the RPMC trial was shown (Fig. 3c). To complete these tasks, participants required complete auditory information, including both sums of the calculation (e.g., “63 + 35”) and all words of the BOSU (e.g., cinema – radio – donkey – newspaper). This means that when the participants saw the current RPMC trial, they had heard all the information necessary to solve the problem. They had to solve the RPMC trial and the concurrent dual-task calculation or BOSU simultaneously. Therefore, calculation and BOSU potentially interfered with the cognitive movement selection period.

Then, participants raised their hand from the response pad, turned the dowel, and simultaneously named the activity or the object, or the solution to the calculation or BOSU. After stating the dual-task solution and having aligned the dowel with the target location, participants placed their hand back on the response pad and the experiment proceeded with the next trial.

#### Subjective difficulty

After the RPMC experiment, participants indicated their subjective difficulty for the dual-tasking conditions, as well as for the plan- and rule-based approaches, on a scale from 1 (very easy) to 10 (very difficult).

### Analysis

Data was analyzed with R version 4.3.2^35^ and RStudio 2023.12.1^36^, running on a Windows 11 PC.

#### Preprocessing and analysis of HRV

Due to technical error, there were no HRV data for three participants, leaving *n*= 45 participants for analyses involving HRV. RR intervals (i.e., the distance between two R peaks in ms) were sampled at a rate of 1000 Hz. Relevant periods were manually annotated during the experiment (i.e., baseline, single-task, activity dual-task, object dual-task, BOSU dual-task or calculation dual-task). Data were stored online on an iPad and then transferred to a PC, where it was analyzed using the RHRV package^37^. The R code and procedure for data cleaning and HRV preprocessing were adapted from the method described by Benz, et al.^38^. Following the approach published by Benz, et al.^38^, RR data were first preprocessed. Artifacts were removed, and missing beats were interpolated. Data was then divided into 17 relevant segments, including the baseline, eight single-task RPMC blocks and eight dual-task RPMC blocks (two per dual-tasking type). For the calculation of HRV, the middle 180 s of each segment were used; if the segment was shorter, the entire block was considered. The root mean square of successive differences (RMSSD) was calculated per segment, and the mean RMSSD for single- and dual-task blocks were calculated per subject. As indicated by the Shapiro-Wilk test, the RMSSD of the different segments was not normally distributed (*p* < .001). Therefore, the non-parametric Wilcoxon signed-rank test was conducted to compare the overall mean RMSSD between single- and dual-tasks. A Friedman ANOVA was conducted to compare the mean RMSSD between the different dual-tasking types.

#### Analysis of secondary task performance

Responses to the secondary tasks - activity-naming, object-naming, BOSU and calculation - were recorded during the dual-tasking condition with the RPMC experiment and in a single-task condition. Accuracy was measured by calculating the percentage of correct answers. According to the Shapiro-Wilk test, accuracy scores for secondary tasks were not normally distributed (*p* < .049). Therefore, accuracy was compared between the single-task condition and the rule- and plan-based movement selection dual-tasks using Friedman’s ANOVA, followed by pairwise Wilcoxon signed-rank tests.

#### Preprocessing and analysis of RPMC data

To evaluate behavioral performance in tasks solved according to the rule- versus plan-based approach and their variability under different dual-tasking conditions, Balanced Integration Scores (BIS)^39^ and movement time were compared between the single- and dual-tasking conditions. Movement time was recorded in milliseconds (ms) during the experiment and could be directly used for analysis. Reaction times and error rates were summarized in BIS. The BIS equally weights reaction times and error rates. It is calculated by subtracting the z-standardized reaction times from the z-standardized error rates. Higher BIS indicates better performance, meaning shorter reaction times and fewer errors. The efficiency advantage measures the superiority of the rule- over the plan-based approach and is calculated by subtracting BIS of the plan-based approach from BIS of the rule-based approach. This procedure was conducted for single-task BIS and for the overall (i.e., mean) dual-task BIS, as well as for each of the dual-task conditions.

The Shapiro-Wilk test indicated that the efficiency advantages and BIS of trials solved using the plan- and rule-based approaches were not normally distributed, in any of the single- or dual-task conditions (*p* < .01), as well as most of the movement time variables (*p* < .05), except for trials in the calculation condition solved using the plan- (*p* = .07) and rule-based approach (*p* = .06). Consequently, the non-parametric Friedman’s ANOVA and Wilcoxon signed-rank test were used to compare the mean BIS and movement time between plan- and rule-based trials in single- and dual-task conditions, and to compare efficiency advantages between single- and dual-task.

If not stated differently, p-values were adjusted for multiple comparisons using the Bonferroni-Holm procedure. Information on reaction time and error rates is given in Table S1.

#### Exploratory regression analyses

Lastly, it was explored which variables would predict the performance in the rule- and plan-based approaches in the different dual-tasking conditions. In order to deconstruct the underlying mechanisms of the rule- and plan-based approaches, eight hierarchical regression analyses predicting the performance in the rule- or plan-based approach in each of the four dual-tasking conditions were conducted. As a first predictor, the single-task performance in the rule- or plan-based approach was entered into the model. This predictor was entered first based on the theory that there might be individual predispositions for plan- or rule-based approaches, or in other words, individual predispositions for the rule-based efficiency advantage, as suggested by Scheib, et al.^5^. Next, the RMSSD during the specific dual-tasking condition was entered in the model, based on the findings reported by Stoll, et al.^6^ that suggested RMSSD, an indicator of parasympathetic nervous system (PNS) activity, affects the plan-based approach specifically. Lastly, in the third step variables that reflect the task difficulty were entered. Therefore, the subjectively perceived difficulty of the task, the mean length of the answers (spoken word in letters), and the mean correctness of the given answers were included.

## Results

### Heart rate variability (RMSSD)

RMSSD was analyzed to determine whether single- or dual-task blocks differed in PNS activity. Mean RMSSD was significantly higher for single- (*Mdn* = 35.31) than for dual-tasking blocks (*Mdn* = 34.86, *p* = .024, *r*=-.24), indicating increased stress (i.e., showed reduced PNS activity) during the dual-tasking blocks.

The RMSSD did not differ between the various dual-tasking conditions (χ^2^(3) = 2.15, *p* = .54; Figure S1, supplementary material).

### Behavioral data

#### Balanced integration scores

To test the dual-mechanisms hypothesis, it was analyzed whether the rule-based efficiency advantage was affected differently by varying dual-task conditions.

**Fig. 4:**
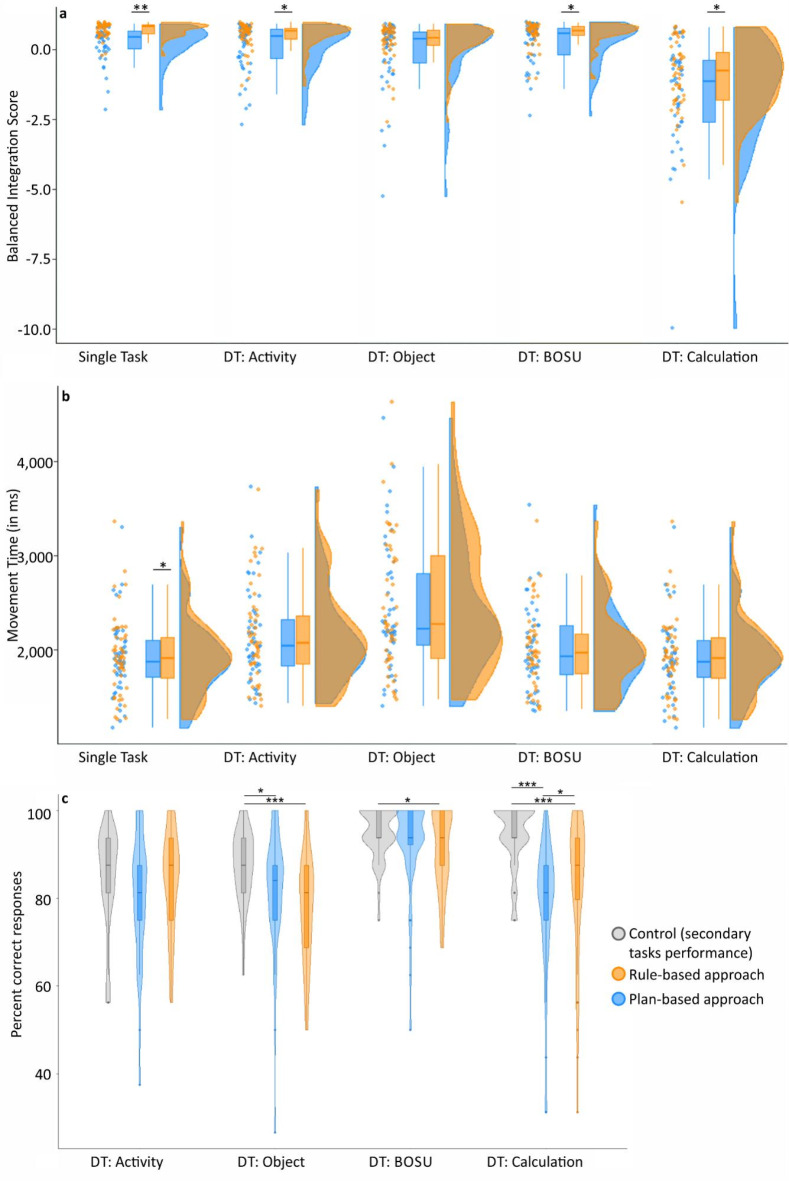
Mean (**a**) BIS and (**b**) movement time for trials solved according to the rule- and plan-based approach per single- and dual-task condition. BIS and Movement Time are displayed for the rule- (orange) and plan-based approach (blue) per single- and dual-task condition. Displayed are raincloud-plots, including the distribution of the data (overlapping for plan- and rule-based approach), jittered raw data points and a boxplot per variable. (**c**) Response accuracy in secondary tasks time-sharing with the rule- (orange) and plan-based (blue) movement selection and in the control situation (gray). *p < .05, **p < .01, ***p < .001.

BIS significantly differed between the plan- and rule-based approaches in the single-task condition and in the dual-task conditions activity, BOSU, and calculation, reflecting the known rule-based efficiency advantage and demonstrating its robustness against various competing secondary tasks. However, BIS of plan-and rule-based approaches did not differ in the object-naming dual-task condition. Results are presented in Table 2; Fig. 4a.

**Table 2:** Comparisons of rule- versus plan-based approach BIS and movement time within conditions by use of Wilcoxon signed rank tests.

Condition	Plan-based approach		Rule-based approach		V	*p*_adj__._	*r*
	Median	Range	Median	Range			
BIS (*N* = 48)							
Single-Task	0.43	−2.15; 0.92	0.81	−0.23; 0.97	139	0.004	− 0.51
Activity	0.46	−2.70; 0.91	0.66	−1.30; 0.97	334	0.017	− 0.27
Object	0.39	−5.25; 0.94	0.44	−2.58; 0.93	477	0.260	− 0.12
BOSU	0.58	−2.37; 1.00	0.69	−1.04; 0.98	327	0.020	− 0.28
Calculation	−1.12	−9.97; 0.82	− 0.76	−5.46; 0.84	311	0.015	− 0.29
Movement Time (*N* = 48)							
Single-Task	1871	1176; 3308	1914	1262; 3363	333	0.041	− 0.27
Activity	2040	1435; 3736	2071	1401; 3705	528	> 0.999	− 0.06
Object	2220	1404; 4465	2273	1471; 4633	457	0.730	− 0.14
BOSU	1932	1355; 3544	1973	1370; 3370	532	> 0.999	− 0.06
Calculation	2019	1351; 3394	2017	1365; 3446	613	0.803	− 0.03

p-Values have been adjusted according to the Bonferroni-Holm procedure.

The rule-based efficiency advantage, defined as the difference in BIS between the rule- and plan-based approaches, did not differ across the dual- and single-task conditions (χ^2^(4) = 3.88, *p* = .422).

However, performance in the plan-based approach varied between conditions (χ^2^(4) = 59.98, *p* < .001), as did performance in the rule-based approach (χ^2^(4) = 100.12, *p* < .001). Follow-up comparisons between the single-task condition and all dual-task conditions revealed that the plan-based performance in the calculation condition was significantly weaker than in the single-task condition (V = 49, *p*_*adj*_.<0.001, *r*=-.65). Performance in the rule-based approach was weaker in all dual-tasking conditions, this means calculation (V = 13, *p*_*adj*_.<0.001, *r*=-.73), BOSU (V = 384, *p*_*adj*_.=0.036, *r*=-.21), activity- (V = 213, *p*_*adj*_.<0.001, *r*=-.41) and object-naming (V = 133, *p*_*adj*_.<0.001, *r*=-.51).

#### Movement time

To examine differences in movement time between the plan- and rule-based approaches, comparisons were made between approaches. Results showed that movement time differed between the plan- and rule-based approaches exclusively in the single-task condition, where the plan-based approach had a significantly shorter movement time than the rule-based approach (see Table 3; Fig. 4b).

#### Performance in secondary tasks

A within-subjects comparison was run comparing accuracy in the single presentation of the secondary task with accuracy when presented in the dual-task condition (combined with rule-based or plan-based movement selection task). Performance in the secondary tasks is shown in Fig. 4c and detailed in supplementary Table S2. Accuracy in these tasks differed significantly between single-task and dual-task conditions (with either rule- or plan-based movement selection) in object-naming (χ^2^(2) = 14.98, *p* < .001), BOSU (χ^2^(2) = 6.41, *p* = .041) and calculation (χ^2^(2) = 37.53, *p* < .001). Follow-up pairwise comparisons revealed that object-naming accuracy was significantly higher in single-task than during both plan- (V = 252 ,*p*_*adj*_.=0.024, *r*=-.26) and rule-based movement selection (V = 70, *p*_*adj*_.<0.001, *r*=-.44). Solving BOSU was significantly worse during rule-based movement selection (V = 136.5, *p*_*adj*_.=0.027, *r*=-.27) compared to single-task. Calculation accuracy was solved best during single presentation (V < 85.5, *p*_*adj*_.<0.001, *r*>-.46) and significantly lower during plan-based than during rule-based movement selection (V = 225, *p*_*adj*=_0.034, *r*=-.22).

### Exploratory analysis of predicting factors

This analysis aimed to identify variables predicting performance in trials solved using the rule- and plan-based approaches under different dual-tasking conditions. The predictors included individual predisposition (single-task performance in BIS), PNS activity (RMSSD), subjective task difficulty, response word length, and response accuracy. Data from 45 participants with RMSSD data were analyzed.

Of the four regression models predicting performance in the plan-based approach, only the model for the activity-naming condition (Table 3) was significant. The final model explained 25.15% of variance in the plan-based performance. Only the word-length of the response significantly predicted plan-based performance, indicating a decreasing plan-based performance with an increasing number of letters. The other predictors did not significantly contribute to the model.

**Table 3:** Regression models predicting the performance in the plan- and rule-based approach in the different dual-tasking conditions.

	ΔR^2^	B	SE B	β	*p*
*Performance in the plan-based approach*					
DT: Activity naming					
Step 1 F(1, 43) = 3.42, *p* = .071	0.074				
Constant		0.004	0.130		0.975
ST-Performance		0.360	0.195	0.271	0.071
Step 2 F(2, 42) = 1.67, *p* = .200	0.000				
Constant		0.001	0.307		0.997
ST-Performance		0.360	0.198	0.271	0.076
RMSSD		0.000	0.007	0.002	0.991
Step 3 F(5, 39) = 2.62, *p* = .039	0.178				
Constant		0.322	1.216		0.793
ST-Performance		0.367	0.189	0.276	0.059
RMSSD		0.001	0.007	0.023	0.871
Difficulty		−0.109	0.069	−0.228	0.125
Word-length		−0.158	0.073	−0.329	0.036
Correctness		2.366	1.347	0.271	0.087
*Performance in the rule-based approach*					
DT: Activity naming					
Step 1 F(1, 43) = 26.75, *p* < .001	0.383				
Constant		−0.310	0.165		0.067
ST-Performance		1.158	0.224	0.619	< 0.001
Step 2 F(2, 42) = 18.89, *p* < .001	0.090				
Constant		−0.837	0.250		0.002
ST-Performance		1.412	0.230	0.755	< 0.001
RMSSD		0.009	0.003	0.330	0.010
Step 3 F(5, 39) = 7.17, *p* < .001	0.005				
Constant		−1.082	0.640		0.099
ST-Performance		1.424	0.239	0.762	< 0.001
RMSSD		0.009	0.004	0.326	0.017
Difficulty		0.012	0.038	0.042	0.730
Word-length		0.018	0.035	0.065	0.607
Correctness		−0.029	0.652	−0.006	0.964
DT: Object naming					
Step 1 F(1, 43) = 17.35, *p* < .001	0.288				
Constant		−0.803	0.269		0.004
ST-Performance		1.517	0.364	0.536	< 0.001
Step 2 F(2, 42) = 77.76, *p* < .001	0.071				
Constant		−1.415	0.383		< 0.001
ST-Performance		1.805	0.374	0.638	< 0.001
RMSSD		0.010	0.005	0.286	0.036
Step 3 F(5, 39) = 5.31, *p* < .001	0.046				
Constant		−1.925	1.193		0.115
ST-Performance		1.714	0.379	0.606	< 0.001
RMSSD		0.009	0.005	0.238	0.101
Difficulty		0.035	0.071	0.067	0.624
Word-length		−0.053	0.049	−0.139	0.281
Correctness		1.802	1.157	0.205	0.127
DT: BOSU					
Step 1 F(1, 43) = 7.45, *p* = .009	0.148				
Constant		0.100	0.178		0.574
ST-Performance		0.657	0.240	0.384	0.009
Step 2 F(2, 42) = 7.64, *p* = .001	0.249				
Constant		−0.389	0.251		0.128
ST-Performance		0.870	0.240	0.509	0.001
RMSSD		0.009	0.003	0.367	0.012
Step 3 F(5, 39) = 3.43, *p* = .011	0.038				
Constant		−4.468	3.154		0.165
ST-Performance		0.918	0.260	0.537	0.001
RMSSD		0.008	0.003	0.352	0.020
Difficulty		−0.022	0.040	−0.080	0.580
Word-length		0.861	0.613	0.221	0.168
Correctness		−0.870	0.990	−0.154	0.385

ST-Performance = performance in single-task condition; RMSSD = mean RMSSD in the respective dual-tasking blocks; difficulty = subjectively experienced difficulty of the secondary task; Word-length = average length of responses given in the secondary task; correctness = correctness of responses given in the secondary task.

Three of four models predicting performance in the rule-based approach were significant: activity-naming (explaining 47.89% of variance), object-naming (explaining 40.51% of variance) and BOSU (explaining 30.56% of variance). In all three models, higher single-task BIS significantly predicted better performance in the rule-based approach. In the activity and the BOSU dual-task conditions, a better performance in the rule-based approach was associated with higher RMSSD. Details of these models are provided in Table 3 (for an overview see supplementary Tables S3-S10).

The evaluation of assumptions for the regression analyses indicated that outliers, independence of errors and multicollinearity were not concerns in these four models. However, the residuals of the three models predicting performance in the rule-based approach deviated from normality (supplementary Figures S3-S5), suggesting caution in generalizing the findings. Detailed information on the assumptions is provided in the supplementary material. Residuals for the plan-based model did not deviate from normality (supplementary Figure S2).

## Discussion

This study investigated whether the superiority of the rule-based over the plan-based approach to movement selection is supported by the dual-mechanisms hypothesis (indicating distinct underlying mechanisms) or by the efficiency hypothesis (suggesting shared mechanisms, but with the rule-based approach using them more efficiently). A dual-task design was employed to explore this question, with rule- and plan-based approaches tested as single-tasks or paired with various secondary tasks (inferring objects with categories or activities from object-interaction sounds, solving calculations, or completing the semantic test BOSU).

### Main findings

The dual-task conditions appeared more stressful than the single-task condition, as indicated by the reduced HRV (RMSSD) in the overall dual-tasking conditions.

Contrary to the efficiency hypothesis, which suggests that load-susceptibility is specific to the plan-based approach, there was no general increase in the rule-based efficiency advantage under dual-tasking conditions. Instead, different secondary tasks had varying impacts on the rule- or plan-based approaches, supporting the dual-mechanisms hypothesis.

### Support for the dual-mechanisms hypothesis

The dual-mechanisms hypothesis is supported by distinct effects of different secondary tasks on the two approaches.

Contrary to the initial hypothesis, there was no increase in the rule-based efficiency advantage during the calculation dual-task condition, triggered by a decrease in the plan-based performance. Instead, both approaches showed significant performance declines, but the rule-based efficiency advantage remained stable. One may interpret this finding as an indicator that both approaches share similar resources with calculation. As suggested by Zago et al.^24^, mental calculation occupies resources in visuo-spatial working memory, necessary to hold multi-digit numbers. Similarly, navigating one’s hand through space as in the RPMC experiment may also require visuo-spatial resources. Thus, the rule- and plan-based approaches may compete with mental calculation for spatial working memory resources, probably on the codes of processing dimension suggested in the multiple resource model^12^. While this broad factor of visuospatial working memory seems to play an important role in both approaches, evidence for distinct underlying resources was found.

Observations in one specific secondary task in this study strongly support the distinctiveness of the two approaches. Naming and categorizing objects from object-interaction sounds eliminated the rule-based efficiency advantage, as performance significantly declined in trials using the rule-based approach. This finding suggests that the rule-based approach might rely on similar resources as those required for naming and categorizing objects. In light of the multiple resource model^12^, it is plausible that the rule-based approach competes for verbal codes of processing with the object-naming-and-categorizing task, as they may share semantic demands. In line with the initial considerations, this suggests a processing site in temporal areas, as suggested by studies linking object-identification^22^ and processing object words^17^ with temporal regions. Contrary, the BOSU dual-task condition did not show a similar pattern. Against the expectations, the rule-based efficiency advantage was maintained in this condition. This finding is surprising since the BOSU is a test for semantic competencies and was anticipated to impair the rule-based efficiency advantage. We propose that the BOSU requires exclusively categorization, while the object-naming task involves identification processes. These semantic processes might function differently^40,41^ and thus have distinct effects on the rule-based approach. This suggests that identification processes might underlie the rule-based approach: Rule identification (placing the thumb on the side with the color-matching LED) could be interrupted by object-sound identification. Summarized, the study demonstrates that certain tasks interfere exclusively with the rule-based approach, reinforcing the assumption of at least partly distinct underlying mechanisms.

Overall, performance in the secondary tasks was good (mean correctness ≥ 78.78%). However, secondary task performance declined compared to single-task during plan- and rule-based movement selection in object-naming and calculation and during rule-based movement selection in BOSU. Additionally, calculation was significantly better when combined with the rule- than with the plan-based approach. In summary, these findings align with the initial theoretical considerations about underlying mechanisms: BOSU performance decreased during time-sharing with the rule-based approach, suggesting shared semantic resources. The reduced calculation performance especially in the plan-based approach supports the notion of shared resources, potentially in spatial working memory mechanisms. Object-naming appeared equally impaired in combination with both approaches, and no significant performance decline was observed for activity-naming.

The plan- and rule-based approaches were examined under the secondary task of inferring actions from object-interaction sounds. The initial expectations for the activity-naming were less specific, as evidence suggests that processing tool-related action sounds may activate a wide-spread left lateralized network, including frontal, parietal and temporal areas^21^. Thus, it was hypothesized that the activity-naming dual-task might interfere with both, the plan- and the rule-based approaches. The present results showed that the activity-naming dual-task, as all secondary tasks, led to a performance decline in the rule-based approach. It is debatable whether this decline results from specific interference between activity-naming and the rule-based approach. On one hand, it might stem from a general susceptibility of the rule-based approach towards competing, cognitively demanding tasks. On the other hand, performance in the rule-based approach is better and shows less variance than performance in the plan-based approach during the single-task condition, indicating a higher potential for performance deterioration. Thus, the performance decline was found only in the rule-based approach.

Regression analyses indicated that longer responses in the activity-naming task led to a performance decline in the plan-based approach. This finding suggests that inferring activity words may interfere with prospective movement planning. This aligns with predominantly left parietal processing sites suggested for the plan-based approach^3^, overlapping with the left-lateralized network suggested to process action sounds (fronto-parietal cortex^14^, dorsal premotor area, inferior parietal lobe^13^) and action knowledge (parietal cortex^15^). This may, however, only come into effect at a certain response length. According to findings from an EEG study^42^, longer words produce higher brain activation at different time points during reading compared to shorter words. If similar processes apply in the activity-naming task, this suggests sensitivity to timing regarding interference effects with the plan-based approach.

Models predicting performance in the rule-based approach for the BOSU, activity-naming, and object-naming conditions were significant. In contrast to the plan-based approach, performance in the rule-based approach in the single-task condition was a significant predictor of the performance in the dual-task conditions. This indicates that participants seem to have an individual performance level in the rule-based approach (single-task) which is indicative of the performance under different external influences (dual-task conditions), emphasizing the inter-individual stability of the rule-based approach.

A further significant predictor for performance in the rule-based approach for the BOSU and activity-naming conditions was identified: Higher RMSSD, an HRV measure reflecting PNS activation and vagal tone^43^, predicted better performance. This means, the more a participant’s system was relaxed, the better was the performance in the rule-based approach in these dual-task conditions. The positive association of vagally mediated HRV with executive cognitive functions was shown in previous studies^44,45^, and may similarly apply for the rule-based approach. However, this finding is surprising, since the RMSSD has previously been associated with the plan- rather than the rule-based approach^6^. This inconsistency may be attributed to differences in the experimental design: this study predicted performance in various conditions, while Stoll, et al.^6^ focused on predicting the performance change due to stress.

### Limitations

When interpreting the present results, certain limitations should be noted. Despite growing evidence in favor for the dual-mechanisms hypothesis, the resources underlying specifically the rule-based approach remain partially uncovered. The present study suggests involvement of semantic resources, but implies that mere semantic word categorization, as manipulated through the BOSU, does not suffice to impair the rule-based approach. Instead, identifying objects from sounds and their categorization seem to feed from the same resources as the rule-based approach. Additionally, since the rule-based approach is disrupted by the multi-step (object-naming-and-categorization) but not the single-step task (BOSU), it could be speculated that the differential mechanisms may not predominantly differ in neuroanatomical regions (which is in line with Randerath et al.^3^), but in timing. In other words, the rule- and plan-based approaches might recruit similar neural structures but occupy these regions for a different duration or at a different time point during processing. Future studies might address this hypothesis by combining functional neuroimaging with methods allowing fine-grained timely resolutions.

Furthermore, significantly longer movement times were found for the rule- compared to the plan-based approach in the single-task condition. This suggests that movement selection processes might have spilled over from reaction to movement time. Thus, the rule-based efficiency advantage measured in BIS could have been overestimated. However, since BIS also includes error rates besides the reaction time, the impact of this spillover on the rule-based efficiency advantage is likely minimal.

Regarding the regression models predicting the performance in the rule-based approach, it must be noted that the residuals were not normally distributed, raising concerns about the generalizability of the findings. The models did not violate any further assumptions (supplementary material).

Lastly, future studies should incorporate movement selection tasks that are more relevant to everyday life. Currently, it is unclear whether the findings on rule- versus plan-based movement selection apply solely to the simple grasp-to-turn task used in this study or if they generalize to other movement selection scenarios.

## Conclusion

The present study examined whether the efficiency or the dual-mechanisms hypothesis explains the superiority of the rule- over the plan-based approach to movement selection best. The efficiency advantage of the rule-based approach showed remarkable robustness towards external influences in the form of secondary tasks. However, despite certain shared general load, the dual-tasking approach, in particular naming and categorizing objects, indicates that the rule- and plan-based approaches use different mechanisms rather than differing exclusively in degrees of efficiency.

## Electronic supplementary material

Below is the link to the electronic supplementary material.

Supplementary Material 1

Supplementary Material 2

Supplementary Material 3

Supplementary Material 4

## Data Availability

The data was made available with the publication of this article.
