# Supplementary material for: The stability and modulation of if-then rules versus prospective planning in movement selection under dual-tasking conditions
Source: Sci Rep. 2025 Jan 8;15:1341. doi: 10.1038/s41598-024-81630-5 (PMC11711758; doi:10.1038/s41598-024-81630-5)
Supplement: Supplementary file 1 — Supplementary Material 1 [file 41598_2024_81630_MOESM1_ESM.pdf]

Supplementary Information to:

The stability and modulation of if-then rules versus prospective planning in movement-selection under dual-tasking conditions

S.E.M. Stoll[1,2,3], A. Wenzel[1], B. Hitzler[1], & J. Randerath[1,2,4*]

[1]Department of Psychology, University of Konstanz, Konstanz, Germany

[2]Lurija Institute for Rehabilitation Science and Health Research, Kliniken Schmieder, Allensbach, Germany

[3]Department of Developmental and Educational Psychology, Faculty of Psychology, University of Vienna, Vienna, Austria

[4]Outpatient Unit for Research, Teaching, and Practice, Faculty of Psychology, University of Vienna, Vienna, Austria

*Corresponding author. Email address: J_Randerath@hotmail.com

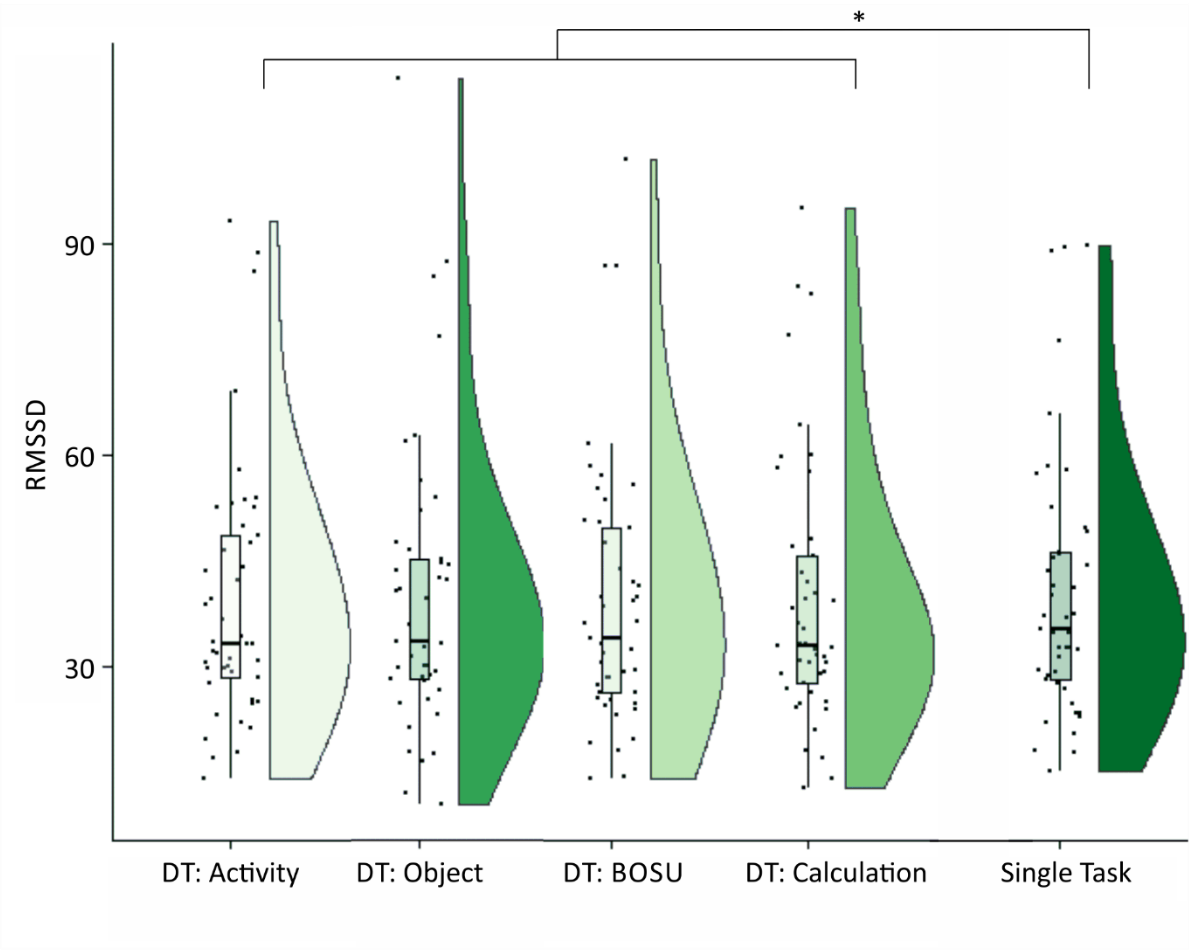

Figure S1. Mean RMSSD per single- and dual-tasking conditions. *$p < .05$. Displayed are raincloud-plots, which include a graphical display of the data distribution, the raw data points and a boxplot per variable.

Condition	Plan-based approach		Rule-based approach		V	$p_{adj.}$	r
	Median	Range	Median	Range			
RT (in ms)							
Single-Task	834	534; 1947	777	525; 1638	983.0	<.001	-.43
Activity	1210	611; 3685	1105	534; 4888	788.0	.160	-.21
Object	1311	586; 5473	1476	601; 7341	505.0	.802	-.09
BOSU	1044	474; 4210	1038	503; 4381	696.0	.819	-.11
Calculation	3457	650; 21513	3423	637; 12868	570.0	.859	-.02
Percent correct trials							
Single-Task	98.5	84.5; 100.0	100.0	94.5; 100.0	69.5	<.001	-.41
Activity	100.0	81.5; 100.0	100.0	91.5; 100.0	5.5	.015	-.29
Object	100.0	69.0; 100.0	100.0	88.0; 100.0	20.5	.078	-.18
BOSU	100.0	87.5; 100.0	100.0	91.5; 100.0	3.0	.036	-.24
Calculation	100.0	77.0; 100.0	100.0	87.5; 100.0	13.5	.012	-.31

Table S1. Pairwise comparisons of reaction times and percent of correct trials between the plan- and the rule-based approach in the single- and all dual-task conditions by use of Wilcoxon signed-rank tests (N=48).

	Median 1	Range 1	Median 2	Range 2	V	$p_{adj.}$	r
**Activity**							
Plan-Rule	81.3	0.38; 1.00	87.5	0.56; 1.00	314.5	.392	-.13
Plan-Control	81.3	0.38; 1.00	87.5	0.56; 1.00	259.0	.119	-.21
Rule-Control	87.5	0.56; 1.00	87.5	0.56; 1.00	325.5	.690	-.04
**Object**							
Plan-Rule	84.0	0.27; 1.00	81.3	0.50; 1.00	601.5	.118	-.16
Plan-Control	84.0	0.27; 1.00	87.5	0.63; 1.00	252.0	.024	-.26
Rule-Control	81.3	0.50; 1.00	87.5	0.63; 1.00	70.0	<.001	-.44
**BOSU**							
Plan-Rule	93.8	0.50; 1.00	93.8	0.69; 1.00	357.5	.479	-.07
Plan-Control	93.8	0.50; 1.00	93.8	0.75; 1.00	126.0	.136	-.19
Rule-Control	93.8	0.69; 1.00	93.8	0.75; 1.00	136.5	.027	-.27
**Calculation**							
Plan-Rule	81.3	0.31; 1.00	87.5	0.31; 1.00	255.0	.034	-.22
Plan-Control	81.3	0.31; 1.00	93.8	0.75; 1.00	36.0	<.001	-.55
Rule-Control	87.5	0.31; 1.00	93.8	0.75; 1.00	85.5	<.001	-.46

Table S2. Pairwise comparisons of secondary task accuracy (in %) between the single-task control condition, the plan- and the rule-based movement selection dual-task conditions by use of Wilcoxon signed rank tests (N=48). p-Values have been adjusted according to the Bonferroni-Holm procedure.

Overview of the regression models. The following tables summarize the results of the regression models that were calculated per approach (plan- vs. rule-based) for each of the dual-tasking conditions, activity, BOSU, calculation, and object.

	**ΔR^2**	**B**	**SE B**	**β**	**p**
Step 1 $F(1, 43)=3.42$, $p=.071$	0.074				
Constant		0.004	0.130		.975
ST-Performance		0.360	0.195	0.271	.071
Step 2 $F(2, 42)=1.67$, $p=.200$	0.000				
Constant		0.001	0.307		.997
ST-Performance		0.360	0.198	0.271	.076
RMSSD		0.000	0.007	0.002	.991
Step 3 $F(5, 39)=2.62$, $p=.039$	0.178				
Constant		0.322	1.216		.793
ST-Performance		0.367	0.189	0.276	.059
RMSSD		0.001	0.007	0.023	.871
Difficulty		-0.109	0.069	-0.228	.125
Word-length		-0.158	0.073	-0.329	.036
Correctness		2.366	1.347	0.271	.087

Table S3. Regression model predicting the plan-based performance in the activity-based dual-tasking. ST-Performance = mean plan-based performance in the single-task blocks; RMSSD = mean RMSSD in the activity-based dual-tasking blocks; Difficulty = subjectively experienced difficulty of the activity-naming task; Word-length = average length of responses given in the activity-naming task; Correctness = correctness of responses given in the activity-naming task.

	ΔR^2	B	SE B	β	p
Step 1 F(1, 43)=1.58, p=.216	0.035				
Constant		-0.038	0.202		.852
ST-Performance		-0.379	0.302	-0.188	.216
Step 2 F(2, 42)=1.03, p=.365	0.011				
Constant		-0.309	0.433		.479
ST-Performance		-0.361	0.304	-0.179	.242
RMSSD		0.007	0.009	0.107	.482
Step 3 F(5, 39)=0.64, p=.667	0.030				
Constant		0.201	2.484		.936
ST-Performance		-0.343	0.312	-0.170	.279
RMSSD		0.008	0.010	0.134	.420
Difficulty		-0.102	0.152	-0.114	.505
Word-length		-0.065	0.104	-0.099	.534
Correctness		1.106	2.469	0.073	.657

Table S4. Regression model predicting the plan-based performance in the object-based dual-tasking condition. ST-Performance = mean plan-based performance in the single-task blocks; RMSSD = mean RMSSD in the object-based dual-tasking blocks; Difficulty = subjectively experienced difficulty of the object-naming task; Word-length = average length of responses given in the object-naming task; Correctness = correctness of responses given in the object-naming task.

	ΔR^2	B	SE B	β	p
Step 1 F(1, 43)=0.105, p=.747	0.002				
Constant		0.250	0.123		.048
ST-Performance		0.060	0.184	0.049	.747
Step 2 F(2, 42)=0.365, p=.697	0.015				
Constant		0.056	0.275		.839
ST-Performance		0.064	0.185	0.053	.731
RMSSD		0.005	0.006	0.121	.434
Step 3 F(5, 39)=0.164, p=.202	0.147				
Constant		-6.940	5.926		.249
ST-Performance		0.085	0.179	0.071	.636
RMSSD		0.007	0.006	0.165	.282
Difficulty		-0.022	0.075	-0.047	.767
Word-length		0.760	1.153	0.114	.514
Correctness		2.888	1.754	0.300	.108

Table S5. Regression model predicting the plan-based performance in the BOSU-based dual-tasking condition. ST-Performance = mean plan-based performance in the single-task blocks; RMSSD = mean RMSSD in the BOSU dual-tasking blocks; Difficulty = subjectively experienced difficulty of the BOSU task; Word-length = average length of responses given in the BOSU task; Correctness = correctness of responses given in the BOSU task.

	ΔR^2	B	SE B	β	p
Step 1 F(1, 43)=2.080, p=.157	0.046				
Constant		-1.736	0.310		<.001
ST-Performance		0.669	0.464	0.215	.157
Step 2 F(2, 42)=1.121, p=.335	0.005				
Constant		-2.018	0.703		.006
ST-Performance		0.682	0.469	0.219	.154
RMSSD		0.007	0.016	0.103	.656
Step 3 F(5, 39)=1.035, p=.411	0.066				
Constant		-35.792	34.498		.306
ST-Performance		0.863	0.483	0.277	.082
RMSSD		0.011	0.016	0.103	.511
Difficulty		-0.164	0.194	-0.132	.403
Word-length		2.621	2.444	0.171	.290
Correctness		-2.749	2.263	-0.192	.232

Table S6. Regression model predicting the plan-based performance in the calculation-based dual-tasking condition. ST-Performance = mean plan-based performance in the single-task blocks; RMSSD = mean RMSSD in the calculation dual-tasking blocks; Difficulty = subjectively experienced difficulty of the calculation task; Word-length = average length of responses given in the calculation task; Correctness = correctness of responses given in the calculation task.

	ΔR^2	B	SE B	β	p
Step 1 F(1, 43)=26.75, p<.001	0.383				
Constant		-0.310	0.165		.067
ST-Performance		1.158	0.224	0.619	<.001
Step 2 F(2, 42)=18.89, p<.001	0.090				
Constant		-0.837	0.250		.002
ST-Performance		1.412	0.230	0.755	<.001
RMSSD		0.009	0.003	0.330	.010
Step 3 F(5, 39)=7.17, p<.001	0.005				
Constant		-1.082	0.640		.099
ST-Performance		1.424	0.239	0.762	<.001
RMSSD		0.009	0.004	0.326	.017
Difficulty		0.012	0.038	0.042	.730
Word-length		0.018	0.035	0.065	.607
Correctness		-0.029	0.652	-0.006	.964

Table S7. Regression model predicting the rule-based performance in the activity-based dual-tasking condition. ST-Performance = mean rule-based performance in the single-task blocks; RMSSD = mean RMSSD in the activity-based dual-tasking blocks; Difficulty = subjectively experienced difficulty of the activity-naming task; Word-length = average length of responses given in the activity-naming task; Correctness = correctness of responses given in the activity-naming task.

	ΔR^2	B	SE B	β	p
Step 1 F(1, 43)=17.35, p<.001	0.288				
Constant		-0.803	0.269		.004
ST-Performance		1.517	0.364	0.536	<.001
Step 2 F(2, 42)=77.76, p<.001	0.071				
Constant		-1.415	0.383		<.001
ST-Performance		1.805	0.374	0.638	<.001
RMSSD		0.010	0.005	0.286	.036
Step 3 F(5, 39)=5.31, p<.001	0.046				
Constant		-1.925	1.193		.115
ST-Performance		1.714	0.379	0.606	<.001
RMSSD		0.009	0.005	0.238	.101
Difficulty		0.035	0.071	0.067	.624
Word-length		-0.053	0.049	-0.139	.281
Correctness		1.802	1.157	0.205	.127

Table S8. Regression model predicting the rule-based performance in the object-based dual-tasking condition. ST-Performance = mean rule-based performance in the single-task blocks; RMSSD = mean RMSSD in the object-based dual-tasking blocks; Difficulty = subjectively experienced difficulty of the object-naming task; Word-length = average length of responses given in the object-naming task; Correctness = correctness of responses given in the object-naming task.

	ΔR^2	B	SE B	β	p
Step 1 F(1, 43)=7.45, p=.009	0.148				
Constant		0.100	0.178		.574
ST-Performance		0.657	0.240	0.384	.009
Step 2 F(2, 42)=7.64, p=.001	0.249				
Constant		-0.389	0.251		.128
ST-Performance		0.870	0.240	0.509	.001
RMSSD		0.009	0.003	0.367	.012
Step 3 F(5, 39)=3.43, p=.011	0.038				
Constant		-4.468	3.154		.165
ST-Performance		0.918	0.260	0.537	.001
RMSSD		0.008	0.003	0.352	.020
Difficulty		-0.022	0.040	-0.080	.580
Word-length		0.861	0.613	0.221	.168
Correctness		-0.870	0.990	-0.154	.385

Table S9. Regression model predicting the rule-based performance in the BOSU-based dual-tasking condition. ST-Performance = mean rule-based performance in the single-task blocks; RMSSD = mean RMSSD in the BOSU dual-tasking blocks; Difficulty = subjectively experienced difficulty of the BOSU task; Word-length = average length of responses given in the BOSU task; Correctness = correctness of responses given in the BOSU task.

	ΔR^2	B	SE B	β	p
Step 1 F(1, 43)=4.011, p=.052	0.085				
Constant		-1.996	0.545		<.001
ST-Performance		1.481	0.740	0.292	.052
Step 2 F(2, 42)=2.928, p=.065	0.037				
Constant		-2.825	0.824		.001
ST-Performance		1.852	0.784	0.365	.023
RMSSD		0.015	0.011	0.206	.190
Step 3 F(5, 39)=2.34, p=.060	0.108				
Constant		-29.218	21.289		.178
ST-Performance		2.278	0.787	0.449	.006
RMSSD		0.016	0.011	0.229	.146
Difficulty		-0.213	0.125	-0.254	.098
Word-length		1.963	1.505	0.191	.200
Correctness		-0.227	1.411	-0.024	.873

Table S10. Regression model predicting the rule-based performance in the calculation-based dual-tasking condition. ST-Performance = mean rule-based performance in the single-task blocks; RMSSD = mean RMSSD in the calculation dual-tasking blocks; Difficulty = subjectively experienced difficulty of the calculation task; Word-length = average length of responses given in the calculation task; Correctness = correctness of responses given in the calculation task.

Assumptions for the regression predicting plan-based performance under activity dual-tasking conditions. It has been reviewed whether all the assumptions of the regression analysis predicting the plan-based performance in the activity-based condition can be assumed.

Outliers and influential cases. There were three cases with standardized residuals larger than |2|, including one case with a very large standardized residual of -3.17. This case also had a covariance ratio of 0.23 which did not lie within the boundaries for covariance ratios (0.6-1.4). However, the leverage values as well as the Cook's distances of all three cases were acceptable. Therefore, it can be assumed that the outliers were no matter of concern in the present analysis.

Assumption of independent errors. The Durbin-Watson test has been used to assess whether the assumption of independent errors has been violated in the present analysis. Results indicated that this was not the case ($DW = 1.69$, $p = .148$).

Multicollinearity. The assumption of no multicollinearity has been investigated with the variance inflation factor (VIF) and the tolerance statistics. We found that multicollinearity was no concern in the model, since the VIF for all predictors were < 1.3 (i.e., well below 10), the tolerance statistics were all > 0.2, and the average VIF = 1.13.

Residuals. Finally, the normality of the distribution of the residuals has been evaluated by a visual inspection of a histogram of the residuals, a qq-plot mapping theoretical and observed values and a scatter plot mapping fitted values and studentized residuals (see figure S1). After visual inspection we concluded that the residuals roughly follow a normal distribution. Moreover, the Shapiro-Wilk test, too, suggested that residuals are normally distributed ($p=.254$).

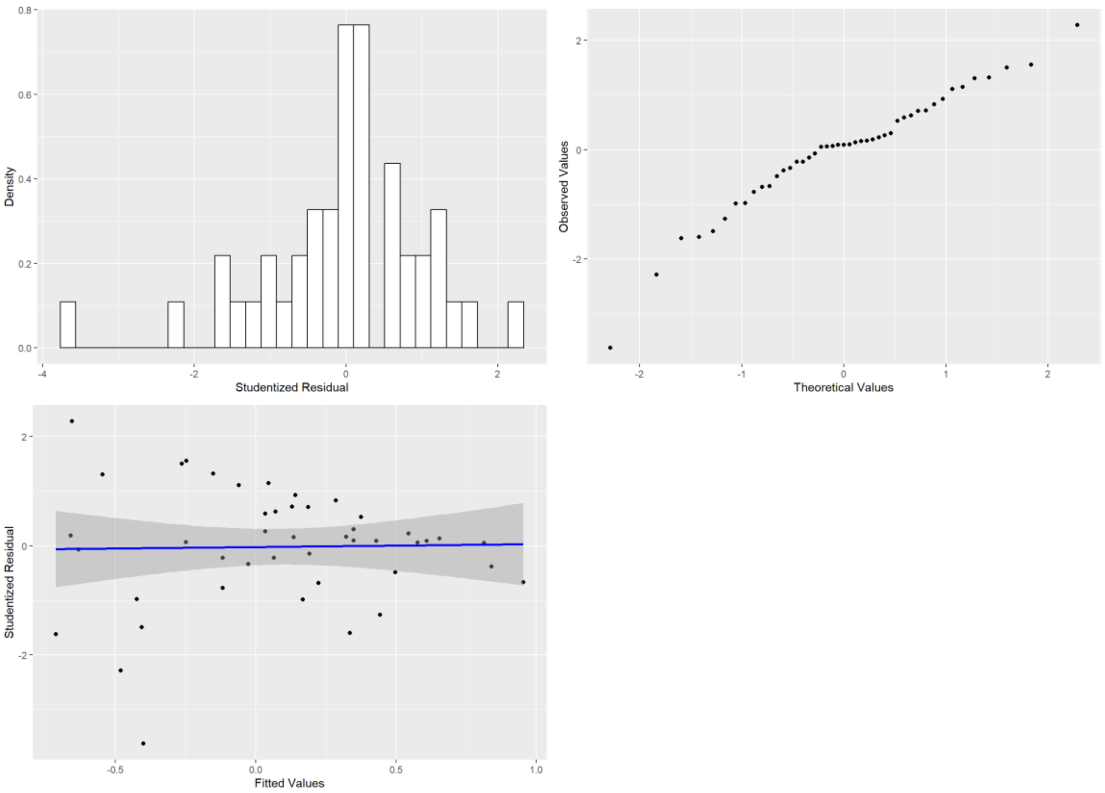

Figure S2. Distribution of the residuals in the regression model predicting the plan-based performance in the activity-based dual tasking condition. Top left: histogram of the studentized residuals. Top right: qq-plot of studentized residuals, mapped on scales of theoretical and observed values. Bottom left: scatter plot of fitted values and studentized residuals.

Assumptions for the regression predicting rule-based performance under activity dual-tasking conditions. It has been reviewed whether all the assumptions of the regression analysis predicting the rule-based performance in the activity-based condition can be assumed.

Outliers and influential cases. There were three cases with standardized residuals larger than |2|, including one case with a very large standardized residual of -3.54. All three cases had covariance ratios which did not lie within the boundaries (0.6-1.4). However, the leverage values as well as the Cook's distances of all three cases were acceptable. Therefore, it can be assumed that the outliers were no matter of concern in the present analysis.

Assumption of independent errors. The Durbin-Watson test has been used to assess whether the assumption of independent errors has been violated in the present analysis. Results indicated that this was not the case (DW = 1.86, p = .341).

Multicollinearity. The assumption of no multicollinearity has been investigated with the variance inflation factor (VIF) and the tolerance statistics. We found that multicollinearity was no concern in the model, since the VIF for all predictors were < 1.3 (i.e., well below 10), the tolerance statistics were all > 0.2, and the average VIF = 1.20.

Residuals. Finally, the normality of the distribution of the residuals has been evaluated by a visual inspection of a histogram of the residuals, a qq-plot mapping theoretical and observed values and a scatter plot mapping fitted values and studentized residuals (see figure S1). After visual inspection we concluded that the residuals do not follow a normal distribution. Moreover, the Shapiro-Wilk test, too, suggested that residuals are not normally distributed (p=.002). This finding might reduce the generalizability of the model.

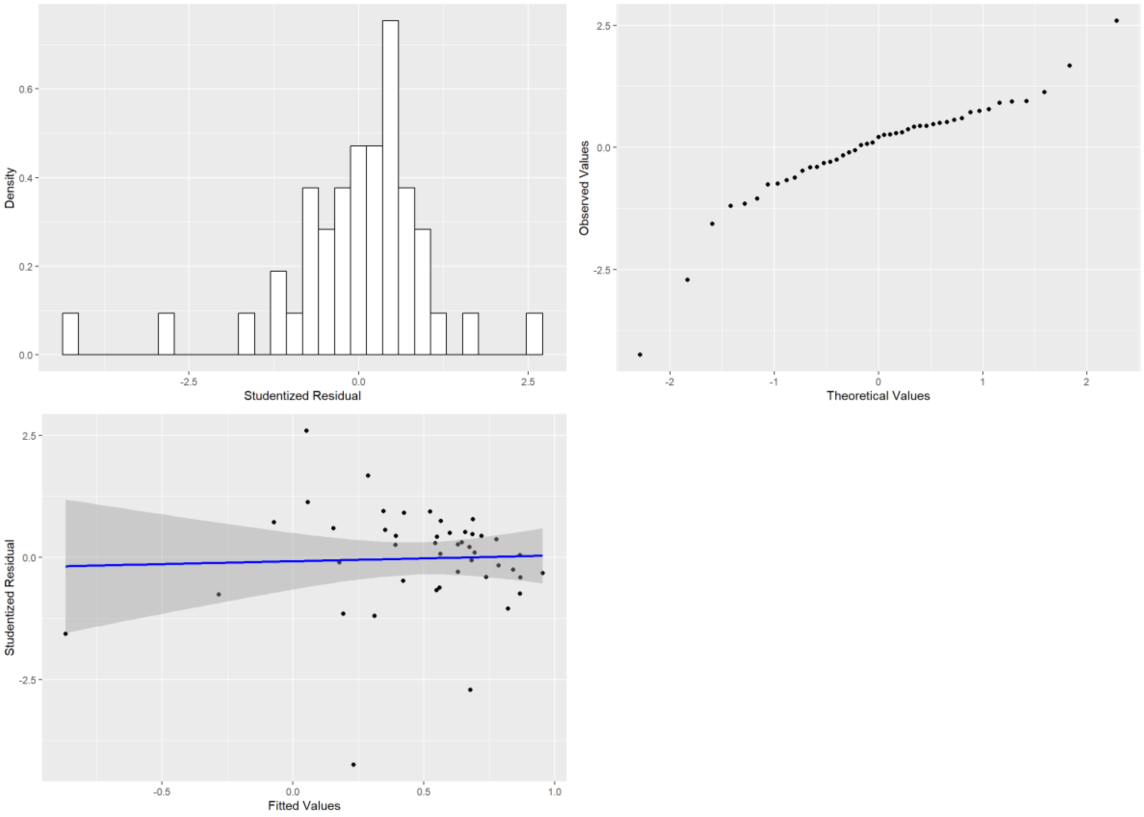

Figure S3. Distribution of the residuals in the regression model predicting the rule-based performance in the activity-based dual tasking condition. Top left: histogram of the studentized residuals. Top right: qq-plot of studentized residuals, mapped on scales of theoretical and observed values. Bottom left: scatter plot of fitted values and studentized residuals.

Assumptions for the regression predicting rule-based performance under object dual-tasking conditions. It has been reviewed whether all the assumptions of the regression analysis predicting the rule-based performance in the activity-based condition can be assumed.

Outliers and influential cases. There were four cases with standardized residuals larger than |2|, including one case with a very large standardized residual of -3.21. Two cases had covariance ratios which did not lie within the boundaries (0.6-1.4). However, the leverage values as well as the Cook's distances of all three cases were acceptable. Therefore, it can be assumed that the outliers were no matter of concern in the present analysis.

Assumption of independent errors. The Durbin-Watson test has been used to assess whether the assumption of independent errors has been violated in the present analysis. Results indicated that this was not the case (DW = 1.95, $p = .423$).

Multicollinearity. The assumption of no multicollinearity has been investigated with the variance inflation factor (VIF) and the tolerance statistics. We found that multicollinearity was no concern in the model, since the VIF for all predictors were < 1.4 (i.e., well below 10), the tolerance statistics were all > 0.2, and the average VIF = 1.18.

Residuals. Finally, the normality of the distribution of the residuals has been evaluated by a visual inspection of a histogram of the residuals, a qq-plot mapping theoretical and observed values and a scatter plot mapping fitted values and studentized residuals (see figure S1). After visual inspection we concluded that the residuals do not follow a normal distribution. Moreover, the Shapiro-Wilk test, too, suggested that residuals are not normally distributed ($p=.035$). This finding might reduce the generalizability of the model.

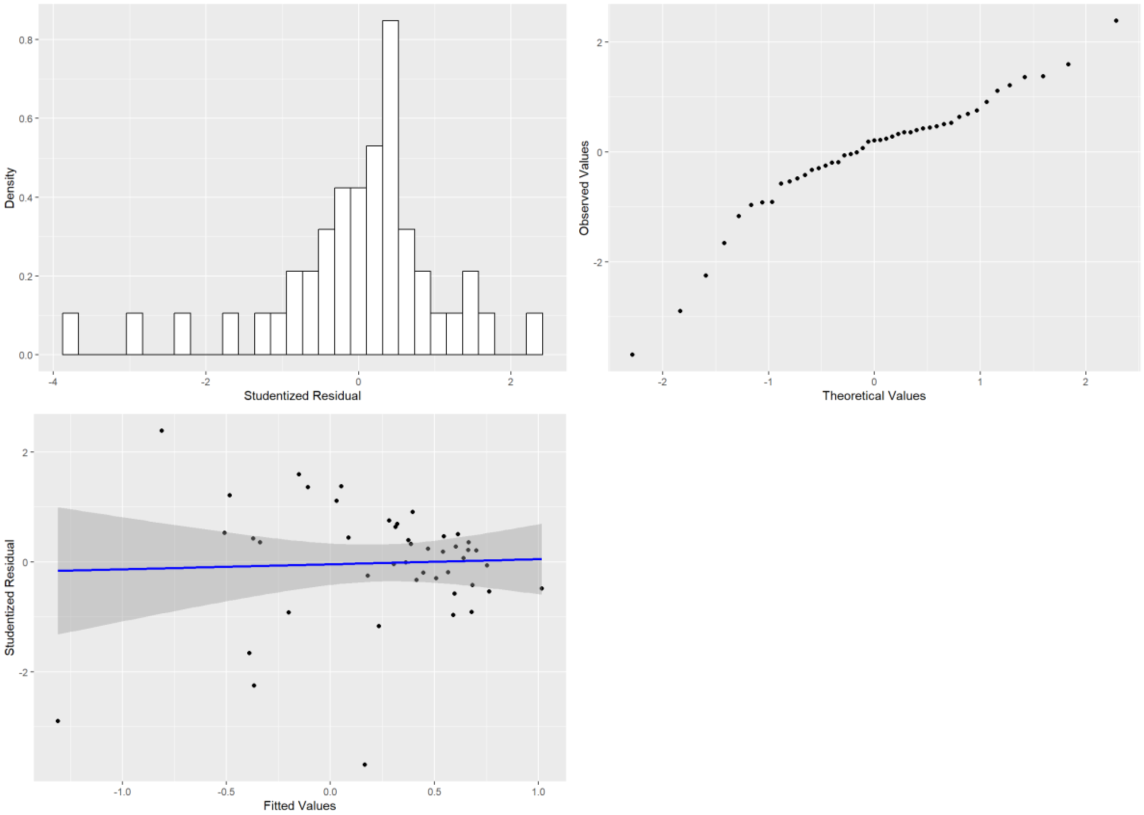

Figure S4. Distribution of the residuals in the regression model predicting the rule-based performance in the object-based dual tasking condition. Top left: histogram of the studentized residuals. Top right: qq-plot of studentized residuals, mapped on scales of theoretical and observed values. Bottom left: scatter plot of fitted values and studentized residuals.

Assumptions for the regression predicting rule-based performance under BOSU dual-tasking conditions. It has been reviewed whether all the assumptions of the regression analysis predicting the rule-based performance in the activity-based condition can be assumed.

Outliers and influential cases. There were three cases with standardized residuals larger than |2|, including one case with a very large standardized residual of -4.13. Two cases had covariance ratios which did not lie within the boundaries (0.6-1.4). However, the leverage values as well as the Cook's distances of all three cases were acceptable. Therefore, it can be assumed that the outliers were no matter of concern in the present analysis.

Assumption of independent errors. The Durbin-Watson test has been used to assess whether the assumption of independent errors has been violated in the present analysis. Results indicated that this was not the case (DW = 1.92, p = .390).

Multicollinearity. The assumption of no multicollinearity has been investigated with the variance inflation factor (VIF) and the tolerance statistics. We found that multicollinearity was no concern in the model, since the VIF for all predictors were < 1.8 (i.e., well below 10), the tolerance statistics were all > 0.2, and the average VIF = 1.35.

Residuals. Finally, the normality of the distribution of the residuals has been evaluated by a visual inspection of a histogram of the residuals, a qq-plot mapping theoretical and observed values and a scatter plot mapping fitted values and studentized residuals (see figure S1). After visual inspection we concluded that the residuals do not follow a normal distribution. Moreover, the Shapiro-Wilk test, too, suggested that residuals are not normally distributed (p<.001). This finding might reduce the generalizability of the model.

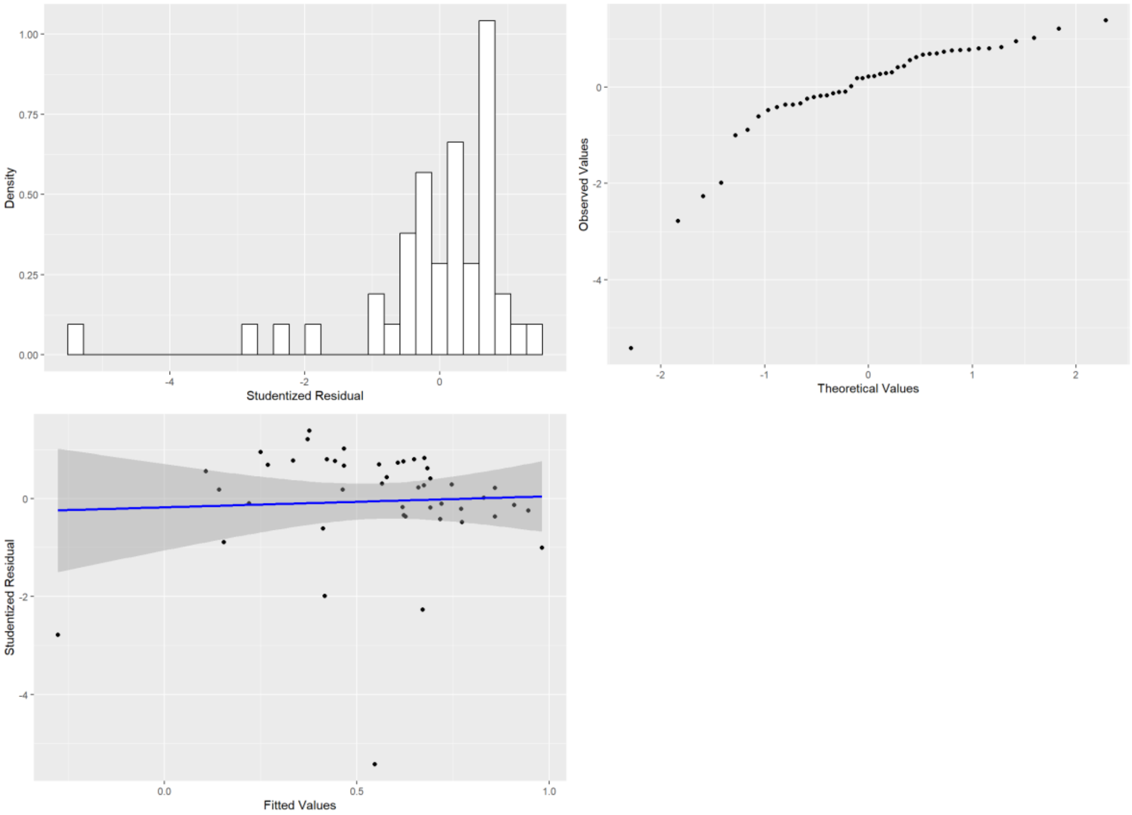

Figure S5. Distribution of the residuals in the regression model predicting the rule-based performance in the BOSU dual tasking condition. Top left: histogram of the studentized residuals. Top right: qq-plot of studentized residuals, mapped on scales of theoretical and observed values. Bottom left: scatter plot of fitted values and studentized residuals.
